# Left Anterior Minithoracotomy for Pulmonary Valve Replacement in Adults

**DOI:** 10.21470/1678-9741-2023-0324

**Published:** 2024-10-18

**Authors:** Alisson Parrilha Toschi, Rodolfo F. Gomes, Renato B. Pope, Mateus B. Bueno, Cézar Suchard, Isaias Cidral, Robinson Poffo

**Affiliations:** 1 Hospital Regional Hans Dieter Schmidt, Joinville, Santa Catarina, Brazil

**Keywords:** Pulmonary Valve, Mitral Valve, Reoperation, Sternotomy, Longevity, Congenital Heart Defects, Postoperative Complications

## Abstract

Surgical interventions on the pulmonary valve in adults have been increasing over the years, as patients with congenital heart diseases are experiencing extended lifespans. Reoperations involving multiple sternotomies exhibit elevated morbidity and mortality rates. With nearly two decades of experience in minimally invasive video-assisted mitral valve surgery, we have chosen the left anterior minithoracotomy approach for addressing the pulmonary valve and right ventricular outflow tract in adult patients. The technique demonstrates safety based on initial outcomes, minimizing potential complications from multiple cardiac reapproaches. Our series of five patients demonstrated an absence of postoperative complications or mortality.

## INTRODUCTION

The pulmonary valve is the most frequently replaced valve in congenital heart diseases. Various conditions may necessitate valve replacement to achieve desired treatment outcomes, including pulmonary stenosis, pulmonary atresia, truncus arteriosus, double outlet right ventricle with pulmonary stenosis, and Tetralogy of Fallot, among others^[1]^.

Pulmonary valve insufficiency is a common complication in the late evolution of patients who underwent surgical correction for Tetralogy of Fallot during childhood. The survival rate into adulthood for patients who have undergone surgical correction of the right ventricular outflow tract exceeds 90%, leading to an increased incidence of late complications related to pulmonary prostheses requiring reintervention^[2]^.

Upon indication of pulmonary valve reintervention, two paths can be pursued: percutaneous or surgical. Both approaches have their technical limitations and risks^[3]^. Overlapping endoprostheses can result in coronary compression, stent fractures, and endocarditis. Median sternotomy carries an increased risk, particularly with repeated occurrences, rendering it inadvisable in certain cases.

Hence, as minimally invasive cardiac surgery techniques were progressively mastered, the use of smaller thoracotomies became routine^[4]^. We began employing the left anterior minithoracotomy, as previously described, as the standard access for pulmonary valve treatment in reoperations and initial surgeries for adult patients.

The following article describes the technique used in cases conducted at this facility, along with the postoperative evolution of the patients.

## SURGICAL DESCRIPTION

Cardioscopic monitoring is established on the patient's back. External defibrillator pads are applied to the upper anterior region of the right hemithorax and the infrascapular area of the left hemithorax. The patient is positioned in a horizontal supine position. Peripheral venous access, central venous access in the left jugular vein, and radial artery access for invasive blood pressure monitoring are punctured.

The patient, under general anesthesia, is placed on mechanical ventilation without the need for selective lung intubation. Transesophageal echocardiography is performed. The patient's body is aseptically prepared, and sterile fields are set up.

The surgery commences with a puncture of the right internal jugular vein and dissection of the right femoral vessels for peripheral extracorporeal circulation establishment.

A skin incision is made in the second or third intercostal (Video 1) space to the left of the sternum, approximately 4 cm in length. Subcutaneous and muscular planes are dissected. The lower rib adjacent to the incision is detached from the sternum to optimize the surgical field. Dissection of the pericardial fat, pericardial sac opening, and exposure of the pulmonary artery trunk are done. Dissection of these structures are performed in cases of reoperation.

**Video 1:**
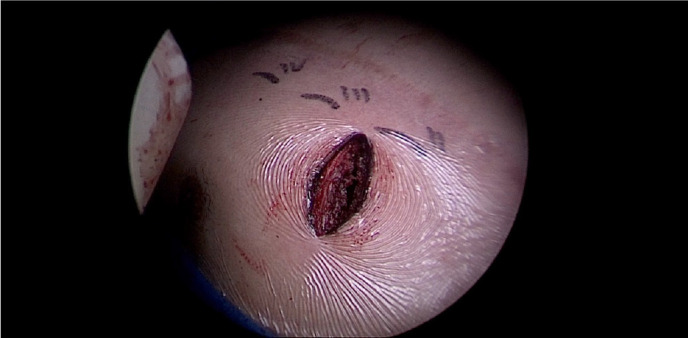
Left anterior minithoracotomy and steps for pulmonary valve replacement.

LInk: https://youtu.be/b9pQc8V33O4

Patient heparinization is then performed at 400 U/kg, followed by cannulation of the femoral vessels and right internal jugular vein using the Seldinger technique, guided by transesophageal echocardiography to centrally position the cannula tips. It is important to note that the inferior venous cannula is advanced to the superior vena cava for proper blood drainage. The vacuum-assisted extracorporeal circulation system is employed in all cases.

Circulatory assistance begins with mild hypothermia until ventricular fibrillation is achieved. This method is chosen for myocardial protection maintenance and optimal bloodless surgical field attainment.

**Fig. 1:**
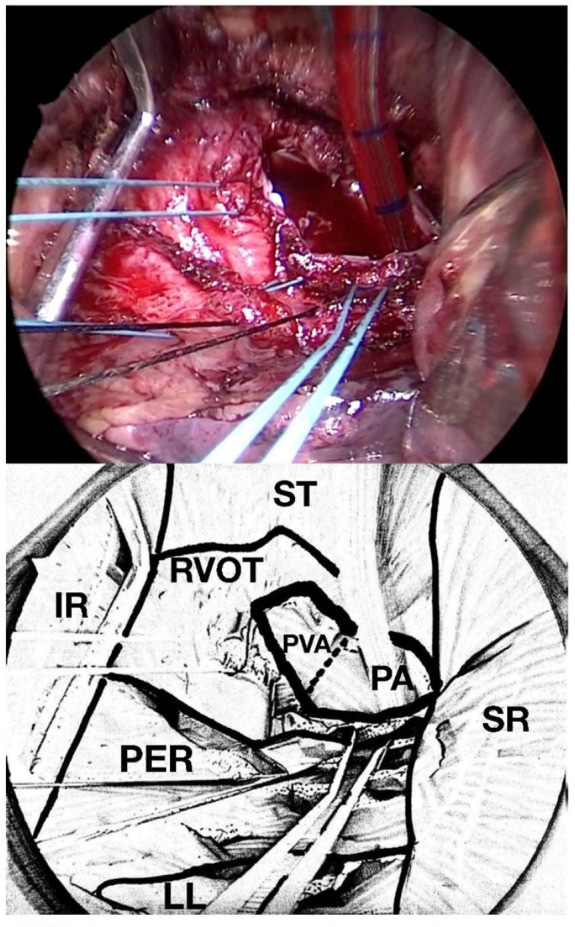
Photo of the operative field and schematic drawing. View of the surgeon positioned to the left side of the patient. IR=inferior rib; LL=left lung; PA=pulmonary artery; PER=pericardial sac; PVA=pulmonary valve annulus; RVOT=right ventricular outflow tract; SR=superior rib; ST=sternum.

A longitudinal incision is made in the native pulmonary artery trunk or the previously implanted pericardium in the right ventricular outflow tract. The incision is extended by 1 cm below the level of the pulmonary valve annulus, expanding the right ventricular outflow tract. Valve leaflets or existing pulmonary prostheses are then excised (Figure 1).

Using separate 2-0 polyester sutures, biological prostheses are anchored in the pulmonary position. These sutures are usually placed in the two-thirds that will be posterior in position on the annulus, enabling the implantation of prostheses with greater effective area. Reconstruction of the right ventricular outflow tract and the pulmonary artery is carried out using continuous 4-0 polypropylene sutures. The anterior third of the valve prosthesis is also affixed to the pericardium using continuous 4-0 polypropylene sutures.

Deaeration of the chambers is performed prior to completing the suturing of the bovine pericardium to the pulmonary trunk. Transesophageal echocardiography monitors this deaeration. The patient is warmed to 37° Celsius. If the heart rhythm is not spontaneously restored, electrical cardioversion is performed. Extracorporeal circulation is weaned. Protamine is administered to reverse heparinization after vessel decannulation.

Drainage of the left pleural space is achieved using a water-sealed chest tube, without closure of the pericardial sac. The rib is reattached to the sternum using number 5 polyester sutures, and the intercostal space is attached with the same type of suture. Other planes are closed conventionally.

## COMMENT

Patients previously operated on for congenital heart diseases, particularly those with Tetralogy of Fallot, have demonstrated a survival rate exceeding 90% over 30 years of follow-up. However, 60.6% of these individuals have remained without the need for reoperation to address pulmonary valve issues^[5]^. Surgically performed pulmonary valve replacement remains the gold standard treatment for these reoperations. Transcatheter treatment of the pulmonary valve accounts for 7.3% of reoperations on the pulmonary valve^[6]^. Minimally invasive surgery aims to reduce morbidity and mortality associated with cardiac surgeries. Reoperations, performed repeatedly through median sternotomies, carry increasing morbidity with each reintervention^[3]^.

With the goal of enhancing the recovery of patients undergoing multiple reoperations, we have initiated the use of the left anterior minithoracotomy for pulmonary valve treatment, as previously reported^[7]^. While other services have increasingly adopted minimally invasive approaches for addressing the pulmonary valve in reoperations^[8,9]^, our use of an anterior minithoracotomy has enabled us to perform pulmonary valve replacement with a suitable surgical field. As in the case described, rib disarticulation was required. This exposure provides access to the pulmonary valve and up to an inch above and below the valve plane. We refrain from rib resection in our surgical procedures.

Our case series includes five patients aged between 20 and 47 years. Among them, three required pulmonary valve re-replacement in the late postoperative period of Tetralogy of Fallot surgical correction, one patient had native pulmonary valve endocarditis, and one patient was at late postoperative period of Ross procedure (Table 1).

**Table 1:** Patients undergoing minimally invasive pulmonary valve replacement.

Patients	Disease	Age (years)	Bypass duration (min.)	Mechanical ventilation (hour)	24-h POB (ml)	BP size
1	T4F	40	74	3.5	550	23
2	T4F	47	120	7.5	300	23
3	T4F	22	117	0	160	23
4	IE	40	119	4.5	100	25
5	ROSS	20	121	3	200	23

BP=biological prosthesis; IE=infective endocarditis; POB=postoperative bleeding; ROSS=Ross procedure late postoperative period; T4F=Tetralogy of Fallot late postoperative period

The indication for mini-incision reoperation in these patients was driven by both pulmonary valve stenosis and insufficiency, along with worsening heart failure and the potential for multiple future reapproaches due to their young age. None of the patients exhibited intracardiac shunts in preoperative examinations. Routine computed aortic angiotomography was requested for peripheral cannulation planning for extracorporeal circulation and selection of the intercostal space to be approached.

No deaths occurred within the group. There were no conversions to sternotomy. The mean extracorporeal circulation time was 110.2 minutes. All patients progressed without incidents in the intensive care units. The mean mechanical ventilation time was 3.7 hours. Low postoperative bleeding was observed in all cases, with an average of 262 ml in the first 24 hours of monitoring. No patient required blood transfusions. All patients continue to receive outpatient follow-up care, classified as functional class I.

The left anterior minithoracotomy proved satisfactory for approaching patients with specific characteristics as aforementioned. However, as this is not a comparative technique study, its safety warrants further investigation with increased statistical power.

## Abbreviations, Acronyms & Symbols

BP: = Biological prosthesis
IE: = Infective endocarditis
IR: = Inferior rib
LL: = Left lung
PA: = Pulmonary artery
PER: = Pericardial sac
POB: = Postoperative bleeding
PVA: = Pulmonary valve annulus
ROSS: = Ross procedure late postoperative period
RVOT: = Right ventricular outflow tract
SR: = Superior rib
ST: = Sternum
T4F: = Tetralogy of Fallot late postoperative period
